# Rocking or Rolling – Perception of Ambiguous Motion after Returning from Space

**DOI:** 10.1371/journal.pone.0111107

**Published:** 2014-10-29

**Authors:** Gilles Clément, Scott J. Wood

**Affiliations:** 1 International Space University, Illkirch-Graffenstaden, France; 2 Lyon Neuroscience Research Center, Bron, France; 3 Azusa Pacific University, Azusa, California, United States of America; McGill University, Canada

## Abstract

The central nervous system must resolve the ambiguity of inertial motion sensory cues in order to derive an accurate representation of spatial orientation. Adaptive changes during spaceflight in how the brain integrates vestibular cues with other sensory information can lead to impaired movement coordination, vertigo, spatial disorientation, and perceptual illusions after return to Earth. The purpose of this study was to compare tilt and translation motion perception in astronauts before and after returning from spaceflight. We hypothesized that these stimuli would be the most ambiguous in the low-frequency range (i.e., at about 0.3 Hz) where the linear acceleration can be interpreted either as a translation or as a tilt relative to gravity. Verbal reports were obtained in eleven astronauts tested using a motion-based tilt-translation device and a variable radius centrifuge before and after flying for two weeks on board the Space Shuttle. Consistent with previous studies, roll tilt perception was overestimated shortly after spaceflight and then recovered with 1–2 days. During dynamic linear acceleration (0.15–0.6 Hz, ±1.7 m/s^2^) perception of translation was also overestimated immediately after flight. Recovery to baseline was observed after 2 days for lateral translation and 8 days for fore–aft translation. These results suggest that there was a shift in the frequency dynamic of tilt-translation motion perception after adaptation to weightlessness. These results have implications for manual control during landing of a space vehicle after exposure to microgravity, as it will be the case for human asteroid and Mars missions.

## Introduction

Human daily activities, such as looking around and walking, involve motions that elicit both tilt and translation of the head. To help maintain an accurate spatial orientation, the central nervous system (CNS) must precisely estimate these head motions. The otoliths of the inner ear act as tiny linear accelerometers that transduce head movements. However, the otoliths signal both head translation and head tilt relative to gravity [1]. The CNS likely resolves this ambiguity through multiple mechanisms, including frequency segregation and multi-sensory integration. Frequency segregation means low frequency linear acceleration sensed by the otoliths is interpreted as tilt while high frequency acceleration is interpreted as translation [2]. Multi-sensory integration suggests that the brain relies on information from other sensors, such as semi-circular canals and vision, to correctly discriminate between tilts and translations [3]–[6]. More specifically, the CNS learns to anticipate a sequence of sensory feedback patterns for any given movement. This mechanism generally involves the use of internal models, or neural representations of physical parameters, and combines efferent and afferent information to resolve sensory ambiguity [7].

In microgravity the otoliths are only stimulated by head translation, not by head tilt. Therefore, the return to normal gravity after spaceflight amplifies the ambiguity between tilt and translation signals [8], [9]. The resulting spatial disorientation can impair human performances during critical mission phases, such as launch and landing. To address this problem, a study was designed to examine both the psychophysical basis and operational implications for tilt-translation disturbances immediately after spaceflight. The objective was to examine the effects of stimulus frequency on the adaptive changes in motion perception during independent and combined tilt and translation motion profiles. One implication of frequency segregation is that there is some region of crossover between low- and high-pass information in which tilt and translation is more difficult to resolve [10]. Multi-sensory integration should be critical around this mid-frequency range. This has been confirmed in studies showing a larger incidence of motion sickness symptoms during off-vertical axis rotation in the 0.2–0.3 Hz range [11], [12]. Because interpretation of otolith signals is complicated at this mid-frequency range, vision and canal inputs are critical for distinguishing between tilt and translation [10]. Testing in darkness and using motion paradigms without canal input in this mid-frequency range will reveal the adaptive mechanisms of otolith processing for motion perception. Therefore, we hypothesized that the perception of tilt would be altered after spaceflight, especially during static tilt and in the low-frequency range of dynamic motion where otolith-mediated responses are greatly reduced in microgravity.

When individuals are rotated at a constant velocity in a centrifuge, they sense the direction of the summed gravitational and centripetal acceleration, i.e. the resultant gravito-inertial acceleration (GIA) vector, as the vertical. Consequently they perceive a roll-tilt of the body when upright. This perception of tilt has been called the *somatogravic illusion* [13]. The perceived body tilt during the somatogravic illusion has been demonstrated to be due to the change in the shear force acting on the utricles [14]. Indeed many experiments have demonstrated that the magnitude of perceived static tilt is the same during actual tilt and during centrifugation [15]–[17].

Perception of static tilt in roll was previously found to be altered after spaceflight [17]–[19]. A 20° overestimation of perceived tilt in roll was observed in seven subjects tilted from 20–90° on the first day after landing compared to baseline values, with a return to normal after two days [20]. Four other subjects also perceived a larger angle of tilt when they were either tilted relative to gravity or exposed to centrifugation following a 16-day Space Shuttle mission [17]. Perception of dynamic roll and pitch tilt was also overestimated after spaceflight during 0.125 Hz Off-Vertical Axis Rotation (OVAR) in 8 crewmembers [21]. During this same experiment, perception of translation during 0.5 Hz OVAR was also overestimated post-flight compared to pre-flight.

In a separate experiment, perception of motion path during sled translation at lower (0.18 Hz) and higher (0.8 Hz) frequencies did not appear to change either during or after spaceflight, although there was an increase in variability of responses [22]. In this latter motion path study, subjects were oscillated along both fore-aft (X-axis) and lateral (Y-axis) directions. Some of the described differenced in results may be attributed to the method of otolith stimulation (tilt versus sled translation), differences in orientation of stimulus, and differences in stimulus frequency. To our knowledge, however, motion perception using both tilt and sled translation around the critical crossover frequency (0.3 Hz, [10]) in crewmembers returning from spaceflight has not been reported before the present study.

The questions asked in this study were the following: (a) Are there changes in perception of tilt in roll and pitch after spaceflight? (b) Are there changes in perception of translation along the Y-axis and X-axis after spaceflight? (c) If so, are these changes frequency-dependent? And (d) Are these changes time-dependent, i.e. how soon after landing does motion perception return to normal? Answers to these questions are relevant for issues such as spatial orientation and manual control following space missions and return to Earth or other planets [23].

## Materials and Methods

### Ethics Statement

This experiment was under taken with the understanding and written consent of each subject. The test procedures were approved by and in compliance with the standards of the NASA Johnson Space Center Institutional Review Board for human testing and were performed in accordance with the ethical standards laid down in the 1964 Declaration of Helsinki.

### Subjects

Eleven subjects (10 males, 1 female), ranging in age from 42–55 years (mean 49 years) participated in this experiment. All were tested before and after eight missions of the Space Shuttle, lasting 11–15 days. All subjects had normal neurological function, as evaluated during the NASA astronaut selection process and subsequent annual medical examination.

### Motion paradigms

This experiment used the NASA variable radius centrifuge (VRC) and the Tilt-Translation System (TTS) located in the Neuroscience Laboratory of the Johnson Space Center in Houston, Texas. Three motion paradigms were utilized in this study: actual tilt about an Earth horizontal axis, fore-aft (X-axis) translation along a linear track, and lateral (Y-axis) translation during constant eccentric rotation. For each of these motion paradigms, subjects could experience various combinations of tilt and/or translation. Therefore, subjects were asked to estimate the magnitude of *both* tilt and translation for each of the three paradigms.

#### Roll-Induced Tilt

Constant velocity variable radius centrifugation was utilized to elicit a perception of tilt (somatogravic illusion) in roll without concordant roll canal cues. Subjects were restrained on a chair that was mounted on a small translation stage fixed to a rotator. A joystick mounted directly in front of the subject was used for reporting motion perception. Data collection was initiated with a slow acceleration (3°/s^2^) to a constant velocity of 216°/s. Subjects continued to rotate for 60 s to allow the post-rotatory response to decay. After the subjects no longer sensed the rotation, the chair was offset by ±6.1, ±12.2, and ±18.5 cm for 5 s corresponding to static roll tilt of the GIA at ±5°, ±10°, and ±15°, respectively. Each static chair position was then maintained for 5 s, and subjects gave verbal reports of amplitude and direction of perceived tilt during the latter half of this period. Subjects then used the joystick to control the chair orientation as part of another experiment (data not reported here).

#### Lateral Translation

While still rotating at 216°/s on the VRC, subjects were oscillated laterally at three discrete frequencies (0.15, 0.3, and 0.6 Hz). The amplitude of the chair translation at each frequency (±11.4, ±9.7, and ±6.1 cm, respectively) was adjusted so that the resultant of translation and centripetal accelerations was ±1.7 m/s^2^, corresponding to tilt of ±10° (Table 1). Subjects reported their perception of roll tilt and/or lateral translation at each frequency using verbal reports (angle of tilt, asymmetry, axis of rotation, amplitude of head motion in space). They also used the joystick to indicate the time of reversal (phase) of the perceived motion.

**Table 1:** Tilt and translation amplitudes and accelerations of the head as a function of stimulus frequency during lateral translation while rotating at 216°/s (VRC 216), and during pure translation (TTS trans) or pure tilt (TTS tilt).

Device	FreqHz	GIA Tiltdeg	Sled Dispm	Sled Accm/s^2^	Centripm/s^2^	GIA Accm/s^2^	Coriolism/s^2^
VRC 216	0.15	10	0.114	0.10	1.63	1.73	0.81
VRC 216	0.3	10	0.097	0.35	1.38	1.73	1.38
VRC 216	0.6	10	0.061	0.86	0.86	1.73	1.73
TTS trans	0.15	10	1.95	1.73	0	1.73	0
TTS trans	0.3	10	0.49	1.73	0	1.73	0
TTS trans	0.6	10	0.12	1.73	0	1.73	0
TTS tilt	0.15	10	0	0	0	1.70	0
TTS tilt	0.3	10	0	0	0	1.70	0
TTS tilt	0.6	10	0	0	0	1.70	0

Freq: frequency; GIA Tilt: tilt of the resultant gravitoinertial acceleration vector relative to the vertical; Sled Disp: peak-to-peak amplitude of sled translation; Sled Acc: peak sled horizontal acceleration; Centrip: peak centripetal acceleration; GIA Acc: peak GIA acceleration; Coriolis: peak linear Coriolis acceleration.

#### Pitch Tilt

Subjects were restrained on the TTS tilt chair that was mounted inside a light-tight enclosure. The tilt motion was provided by dual-wheel friction wheels using direct drive servo motors and a pivoting yoke assembly to provide up to ±20° dynamic displacement. Subjects were restrained in the chair with straps and padding around the shoulders, mid-torso, and waist. The chair height was adjusted so that the head (inter-aural axis) was aligned with the tilt axis, and the head was restrained in an upright orientation. Noise cancelling headphones were used to mask auditory cues. A chair-mounted joystick was used for motion perception. The session started with chair tilts at ±2.5°, ±5°, and ±7.5° (angular velocity approximately 2.5°/s) presented in a random order. Each static chair position was then maintained for 5 s, and subjects provided verbal reports of perception during the latter half of this period.

#### Fore-Aft Translation

The enclosure then translated along an air-bearing track by means of three linear motors operated in series in a single magnet track. In fact, subjects were either translated along the track or tilted in the pitch plane at three discrete frequencies (0.15, 0.3, and 0.6 Hz). The amplitude of the translation at each frequency was adjusted (±195, ±49, and ±12 cm, respectively) so that the acceleration was ±1.73 m/s^2^, equivalent to ±10° of tilt in pitch (see Table 1). Subjects reported their perception of pitch tilt and/or fore-aft translation at each frequency, using verbal reports to indicate amplitude and the joystick to indicate phase. Subjects were instructed “to move the joystick to the perceived tilt angle as though you were controlling the motion”.

### Study Schedule

All astronaut-subjects were tested during three pre-flight sessions at approximately launch minus (L-) 120 days, L-90 days, and L-60 days. Eight astronaut-subjects were first tested with the VRC during roll-induced tilt and lateral translation between 1–4 hours after return to Earth (R+0 day) and with the TTS during pitch tilt and fore-aft translation on the day after landing (R+1 day). These eight subjects and two other subjects were then tested with both devices at R+2, R+4, and R+8 (±1–2 days depending on subject availability). One subject was not tested until R+4 after landing because of general motion sensitivity.

### Data Analysis

We measured the subjective estimates of tilt and translation perception during fore-aft translational and pitch tilt stimuli (TTS) and during lateral translation and centrifugation (VRC). Fore-aft translation gain was defined as the ratio of the perceived amplitude of translation over the actual sled translation. Lateral translation gain was defined as the ratio of the perceived amplitude of translation over the combined translation and centripetal acceleration. Pitch tilt gain was defined as the ratio of the perceived amplitude of tilt over the actual chair tilt. Roll tilt gain was defined as the ratio of the perceived amplitude of tilt over the GIA tilt during centrifugation.

Differential perceptions gain were calculated as the difference between the lateral translation gain and the dynamic roll tilt gain, and as the difference between the fore-aft translation gain and the dynamic pitch tilt gain.

Nonlinear least squares sinusoidal curve fits were used to describe the modulation of the joystick responses as a function of the sinusoidal-varying linear acceleration stimulus using a custom Matlab script (MathWorks, Inc.). The curve fits were used to determine the phase relative to tilt or translation position, with positive leading and negative lagging [24]. Phases for tilt motion were also corrected for 180° differences that occur depending on whether the axis of rotation is above or below the head. These response parameters and the verbal reports were analyzed with repeated measures multiple analysis of variance using a commercial statistics program (SPSS, IBM). Using an alpha error of 0.05 as the decision rule, the null hypothesis that there is no difference across mission day (pre-flight, R+0/1, R+2, R+4, and R+8 days), and stimulus frequency (0.15, 0.3, and 0.6 Hz) was tested with Wilke’s lambda serving as the critical statistic. The same alpha error was used for paired t-tests for comparing specific post-flight parameters relative to the pre-flight averages.

## Results

### Static Tilt

**Figure 1:**
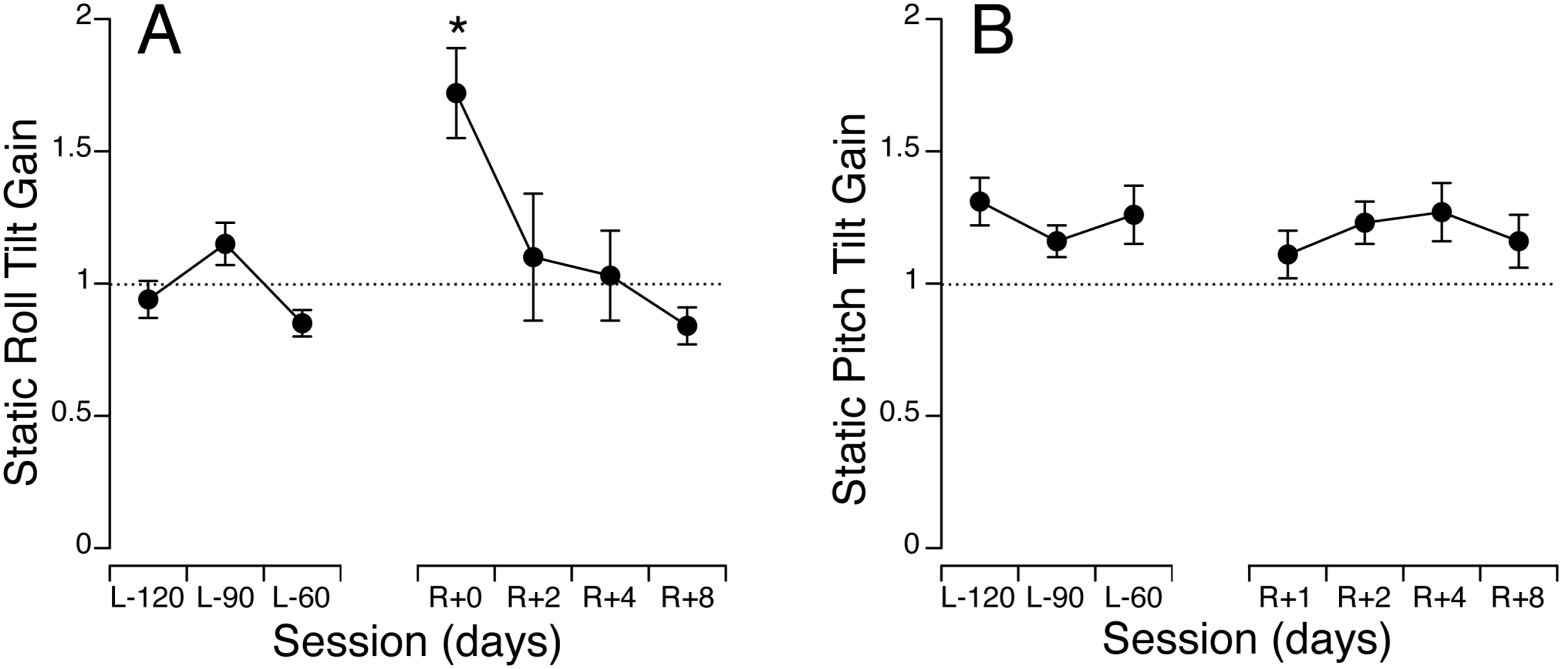
Gain perception during static tilt in roll induced by centrifugation (A) or static tilt in pitch induced by actual tilt relative to gravity (B) before (L-120, L-90, L-60 days) and after (R+0 to R+8 days) spaceflight. In A, gain was calculated as the ratio of subjective reported tilt versus tilt of the gravitoinertial acceleration (GIA) vector. In B, gain was calculated as the ratio of subjective reported tilt versus actual chair tilt. Dashed lines correspond to a gain of unity. Mean ± SE of 10 subjects. *p<0.05 relative to the last pre-flight session.

On Earth, when subjects had to estimate their body tilt angle during centrifugation, i.e. in absence of semi-circular canal stimulation, they perceived accurately the orientation of the GIA (Figure 1A). When the chair was actually tilted relative to gravity, i.e. following semi-circular canal stimulation (Figure 1B), they overestimated their tilt during small true pitch tilt angles (*p*<0.01 relative to a gain of unity), a phenomenon known as the Müller effect [3]. Compared to pre-flight, there was a significant (paired t-test, *p*<0.01) increase in the static roll tilt perception gain on R+0, which returned to baseline values by R+2 days. By contrast, there was no change in the static pitch tilt perception gain after the flight compared to before.

### Sinusoidal Lateral Translation

**Figure 2:**
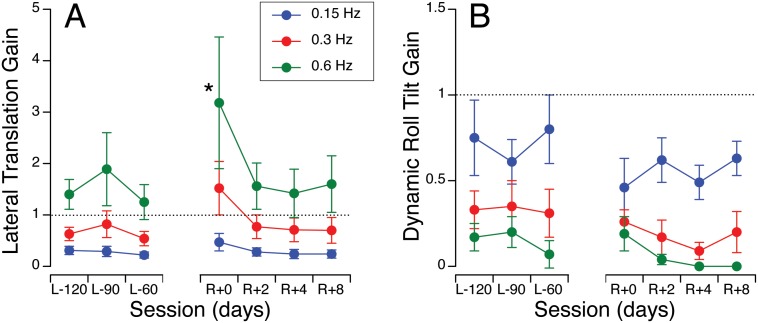
Perception gains during lateral oscillations at 0.15, 0.3, and 0.6 Hz before and after spaceflight. **A.** The reported magnitude of self-displacement sideways was divided by the actual magnitude of the translation stage (translation was actually ±11.4, ±9.7, and ±6.1 cm at 0.15, 0.3, and 0.16 Hz, respectively). **B.** The reported angle of roll tilt was divided by the tilt of the GIA when the translation stage reached maximum eccentricity (GIA tilt was theoretically ±10° at each frequency). Dashed lines correspond to a gain of unity. Mean ± SE of 10 subjects. *p<0.05 relative to the last pre-flight session.

Before flight, when subjects were translated laterally in the VRC in the dark, the perceived magnitude of their displacement was less than their actual displacement for the 0.15 and 0.3 Hz frequencies, i.e. their lateral translation perception gain was lower than unity (Figure 2A). On the other hand the translation perception gain ranged from 1.2–1.8 at the higher frequency (0.6 Hz). Lateral translation gains were analyzed using a 7 (sessions; L-120, L-90, L-60, R+0, R+2, R+4, R+8) ×3 (frequencies; 0.15 Hz, 0.3 Hz, 0.6 Hz) repeated-measures ANOVA. This two-way ANOVA yielded a significant difference in lateral translation gain across test sessions [F (6,189) = 3.32, *p* = 0.004] and frequencies [F (2,189) = 30.2, *p*<0.001]. The variability across subjects was also increased during the early post-flight testing. During the same stimuli, the subjects had a small sense of tilt in roll, with a larger perception gain for the lower frequency (0.15 Hz) than the other frequencies (Figure 2B). There was no significant difference in the dynamic roll tilt gain across test sessions [F (6,189) = 1.85, *p* = 0.09] but there was a significant difference across frequencies [F (2,189) = 49.8, *p*<0.001]. The absence of change in the roll tilt perception gain during sinusoidal motion after spaceflight contrasts with the increase in the roll tilt perception gain seen during static tilt after spaceflight (see Figure 1A).

### Sinusoidal Fore-Aft Translation

**Figure 3:**
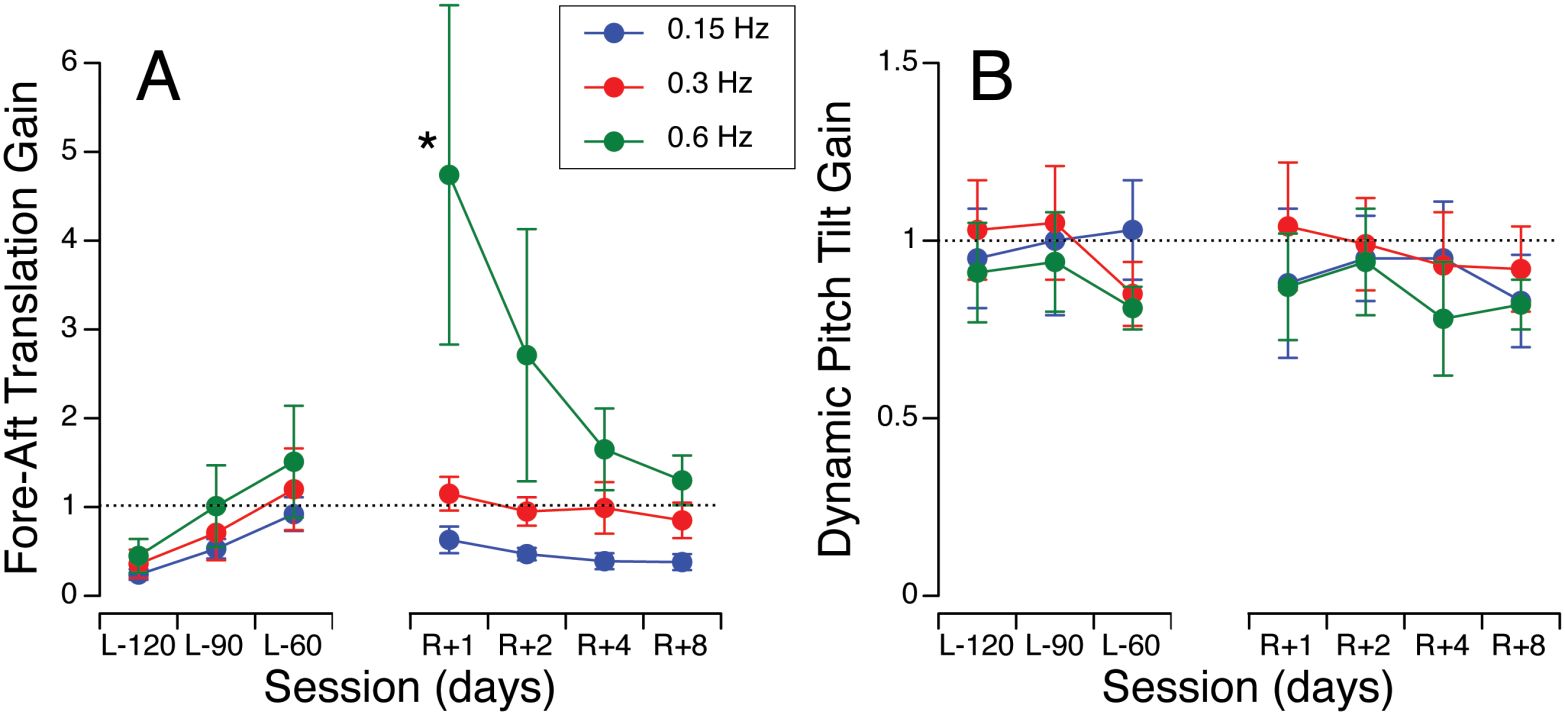
Perception gains during fore-aft oscillations at 0.15, 0.3, and 0.6 Hz before and after spaceflight. **A.** The reported magnitude of fore-aft self-displacement was divided by the actual magnitude of sled translation along the track (translation was actually ±195, ±49, and ±12 cm at 0.15, 0.3, and 0.16 Hz, respectively). **B.** The reported angle of tilt in pitch was divided by the maximum tilt of the chair (tilt varied between ±10° at each frequency). Dashed lines correspond to a gain of unity. Mean ± SE of 10 subjects. *p<0.05 relative to the last pre-flight session.

During sinusoidal fore-aft translation in the TTS’ first session, subjects generally reported very small magnitudes of self-displacement at all frequencies, so their translation perception gain was ranging from 0.1–0.3. When subjects had a second look at the TTS apparatus during their second and third session, they presumably realized that the amplitude of sled displacement could be much larger than what they reported during the first session. This is probably the reason why the translation perception gain increased from the first to the third pre-flight session (Figure 3A). A two-way ANOVA yielded a significant difference in fore-aft perception translation gain across test sessions [F (6,189) = 2.59, *p* = 0.02] and frequencies [F (2,189) = 14.1, *p*<0.001]. The fore-aft perception translation gain significantly increased on R+1 for the highest frequency (paired t-test, *p*<0.05) and then gradually returned to pre-flight baseline. As with lateral translation, the variability in responses also increased during the early post-flight testing.

It is interesting to note that during pure fore-aft translation on the sled, subjects rarely reported any pitch tilt, i.e., hilltop illusion, even though the chair was clearly capable of simultaneously tilting and translating. However, during sinusoidal tilt of the subject in pitch, the reported angle of tilt was similar to that of the actual tilt, hence a gain close to unity for all three frequencies tested. No significant difference in dynamic pitch tilt gain was found across test sessions [F (6,189) = 0.38, *p* = 0.89] nor frequencies [F (2,189) = 1.65, *p* = 0.19] (Figure 3B).

**Figure 4:**
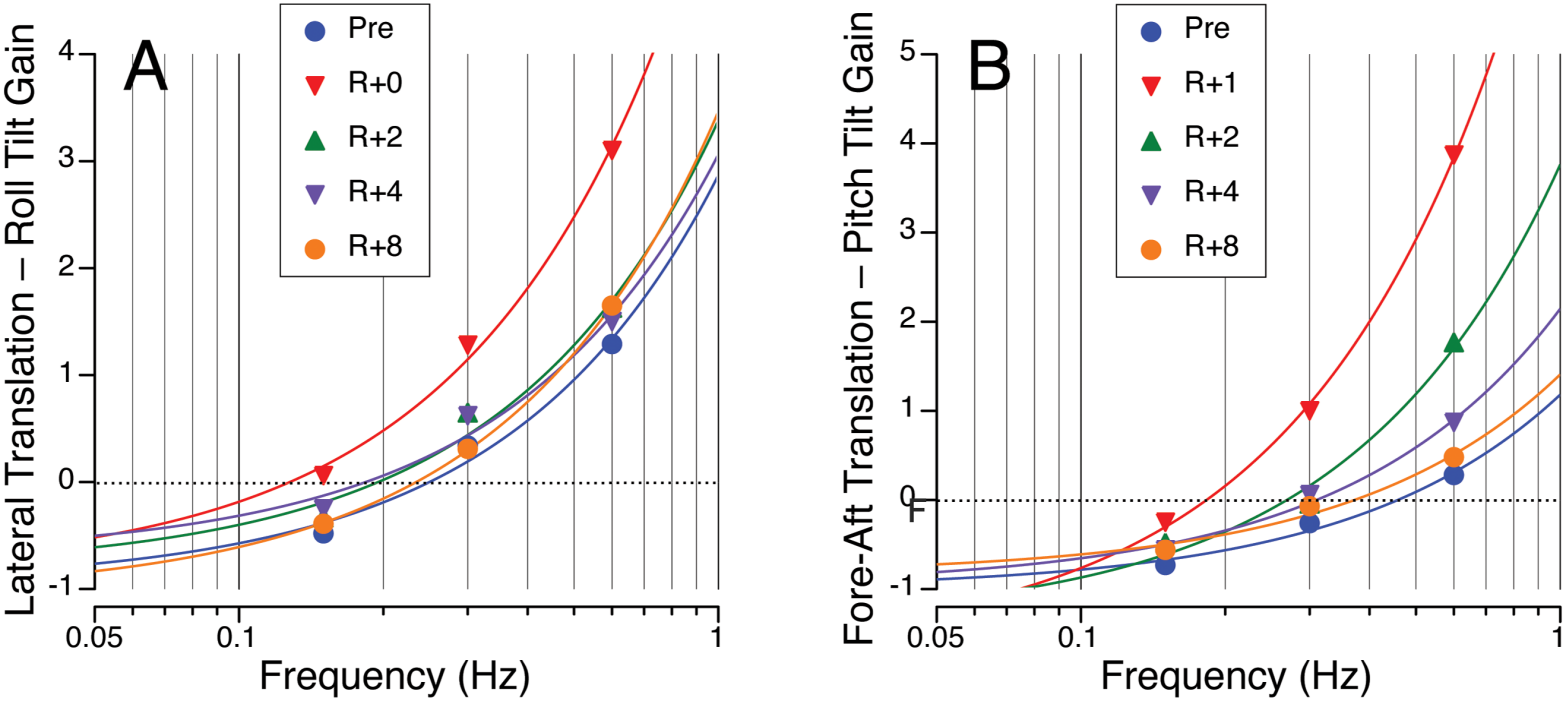
Differences between the translation gain and the tilt gain during dynamic lateral oscillations and roll-induced tilt (A) and during fore-aft oscillations and pitch tilt (B). Data points represent the mean differential gains obtained during translation or tilt at 0.15, 0.3, and 0.6 Hz. Curves are quadratic fits to the data using the least-square regression method (R^2^ are 0.96 or higher). The intercept of each curve fit with the dashed line is the crossover frequency between the perception of tilt or translation: a negative difference (below zero) corresponds to a perceived tilt > perceived translation; a positive difference (above zero) corresponds to a perceived translation > perceived tilt.

By computing differential gains, i.e. the difference between translation perception gain and tilt perception gain, and plotting them as a function of frequencies tested, we could determine at which frequency of stimulation the subjects perceived more tilt versus more translation during each session. This crossover frequency is when the least-squares fit quadratic curves on Figure 4 crosses the zero line, i.e., when the difference between the perception of translation and tilt is zero. Pre-flight, this crossover frequency was 0.25 Hz during lateral translation and 0.45 Hz during fore-aft translation. The consistently higher crossover frequency during fore-aft translation can be attributed to the concurrent canal contributions that were absent during the lateral translation runs. Immediately after return to Earth this crossover frequency was 0.13 Hz during lateral translation and 0.18 Hz during fore-aft translation. This indicates the perception of translation was dominant at lower frequencies post-flight, regardless of the motion paradigm. Recovery of responses during lateral translation was faster (by R+2) than during fore-aft translation (by R+8). After 8 days following landing, the crossover frequency was back to baseline for both paradigms (0.23 Hz during lateral translation; 0.37 Hz during fore-aft translation).

### Which Way Am I Going?

**Figure 5:**
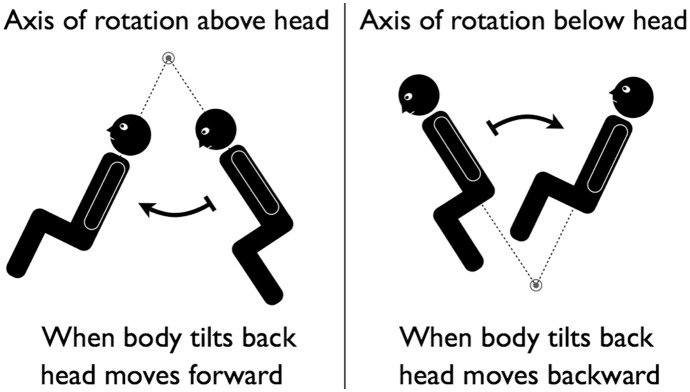
The phase of the subjective fore-aft direction of head motion relative to tilt motion depends from whether the axis of rotation during body tilt in pitch is perceived to be above or below the subject’s head.

In addition to the magnitude of perceived tilt and translation described above, we also asked subjects to use a joystick for indicating the direction in which they were moving. It is interesting to point out that the sensed direction of translation should be opposite for the subjects who perceived the axis of rotation to be above their head compared to those subjects who perceived the axis of rotation to be below their head (Figure 5).

During pure body tilt in pitch, although the axis of rotation of the chair was at the level of the subject’s inter-aural axis, four subjects reported that the axis of rotation was located above their head, and the seven others that the axis was below their head. The subjects were asked to estimate the distance between the axis of rotation and the center of their head. When averaged across subjects and sessions, this estimated distance was either 50.1 cm above the center of the head (SD 19.6 cm), or 26.9 cm below the center of the head (SD 26.4 cm). Interestingly enough, the mean reported translation of the head was 16.7 cm (SD 10.5 cm) or 9.5 cm (SD 9.5 cm), for those subjects who reported the axis of rotation was above or below the center of the head, respectively. A calculation of the theoretical translation for the subject’s estimated distances to the axis of rotation for these radii at ±10° rotation are 17.3 cm and 9.3 cm, for above and below the center of the head, respectively. These values are close to those reported by our subjects. A paired t-test indicated no significant difference between the perceived distance of the axis of rotation across the pre-flight and post-flight sessions.

During sinusoidal lateral oscillations generating a ±10° roll tilt of the GIA, the same four subjects reported that the axis of rotation was above their head. They estimated this distance to be 71.0 cm (SD 67.9 cm) in average, and their translational displacement to be 73.4 cm (SD 98.9 cm). The other subjects reported the axis of rotation to be 98.8 cm (SD 20.9 cm) below their head, i.e., at the seat level, and a perceived peak-to-peak translation of 42.7 cm. In both cases, the perceived translation was larger than predicted from theoretical calculations would predict if the subjects were actually tilted ±10° (24.5 cm and 34.1 cm, respectively). The perceived distance of the axis of rotation was not significantly different across the pre-flight and post-flight sessions.

After accounting for differences in the perceived axis of rotation, the phase of the perceived motion remained close to the actual motion path, with phase errors rarely exceeding 20°. Although the variability was increased during the early post-flight testing for both the fore-aft and lateral translation paradigms, there were no significant changes in phase error between pre-flight and post-flight testing.

## Discussion

### Static Tilt

The perception of static tilt reported by all subjects during eccentric rotation in darkness on the VRC is the classical somatogravic illusion in response to a tilt of the GIA [3]. Our data showing an increase in the static roll tilt perception gain on landing day is in agreement with previous data, which also demonstrated an increase in this illusion immediately after a 16-day space mission [17]. This overestimation of perceived static roll tilt has been attributed to a decrease in the internal estimate of gravity when tilt is no longer sensed by the otoliths in weightlessness [8], [9], [17].

In weightlessness, the gravity signal to the otolith organs is altered. In weightlessness, there is an absence of gravitationally-based otolith stimuli to the CNS, which creates a sensory conflict that appears to be the cause of the disorientation and motion sickness experienced by many astronauts during the first few days of a space mission [25]. However, after several days in orbit these symptoms subside, suggesting that otolith signals are then interpreted unambiguously in microgravity. This reinterpretation then presumably carries over to the post-flight period, which causes an overestimation of tilt when entering a gravitational field again.

By contrast, our results showed no change in the perception of tilt during static pitch post-flight relative to pre-flight. This difference can be most likely attributed to the fact that testing on the TTS took place one day later than testing on the VRC. An earlier TTS session might have given a stronger effect. Indeed posturography tests performed daily after return of the Space Shuttle show significant impairments in equilibrium control on landing day that return to normal within less than one day [26]. Other factors may be that the tilt angles of the stimuli were smaller in pitch than in roll (7.5° versus 15°), and that the otolith and semi-circular canal stimuli were different (tilt versus eccentric rotation). The results of ground-based studies also indicate that the inherent difference between perception of tilt in pitch and roll are not well understood [27].

### Dynamic Tilt and Translation

In both the roll and the pitch axes, the dynamic perception gains reflect a frequency dependent response, with tilt gains greater at the lowest frequency (0.15 Hz) and translation gains greater the highest frequency (0.6 Hz). Consistent with other studies, this frequency dependency is greater when congruent canal cues are absent [10], [16]. In fact, with both canal and otolith cues present during rotation about an earth-horizontal axis in pitch, the tilt perception gains remained close to unity at all frequencies. The lack of changes during sinusoidal pitch tilt during post-flight testing may also be due to increased dependence on canal cues for tilt estimation when these signals are present. Previous flight studies have shown that both the vestibulo-ocular reflex and the perception self-rotation in response to dynamic tilt in pitch were not different in-flight and post-flight from pre-flight [28].

The dynamic roll tilt perception gains were generally less than unity throughout both pre-flight and post-flight testing. During centrifugation, there is no semi-circular canal stimulation and the perception of roll tilt (the somatosensory illusion) is the result of an interpretation by the CNS that the direction of the GIA vector is vertical. Therefore, the sensation of tilt during centrifugation is expected to be smaller than when there is an actual tilt of the body and semi-circular canal stimulation (see Figure 3B). While the dynamic roll tilt perception was significantly greater at 0.15 Hz than at the other frequencies during pre-flight testing, this difference was less during post-flight testing (see Figure 2B).

In both VRC and TTS motion paradigms, the dynamic translation perception gain was significantly increased after spaceflight, especially at the highest frequency. A similar overestimation of translation has also been observed during off-vertical axis rotation (OVAR) following spaceflight [24]. This increase in translation perception gain may suggest that the threshold for translational acceleration profiles is lowered after spaceflight [22]. This result is consistent with the observation that all of our crewmember participants expressed an increased sensitivity to voluntary motion after landing.

### Tilt-Translation Ambiguity

We propose that the greater changes in translation perception gain on R+0 relative to changes in tilt perception suggest that there is a shift in the crossover frequency of tilt and translation perception after spaceflight [10]. Ground-based studies have shown that during pure linear acceleration in the dark, there appears to be a natural segregation of otolith-mediated tilt and translation responses for both eye movements and self-motion perception [16], [29]. The ambiguity of otolith afferent information is the greatest in the frequency region where tilt and translation responses crossover, as shown by the increase in incidence of motion sickness in this region [10], [11], [24]. Consistent with these previous reports, our results showed that perception gain changed as a function of stimulus frequency: tilt perception gain decreased and translation perception gain increased as stimulus frequency increased. Pre-flight, the frequency at which there was a crossover of perceived tilt and translation was 0.25 Hz during lateral translation and 0.45 Hz during fore-aft translation. Since the amplitude of linear acceleration of the head was equivalent across paradigms (peak acceleration equivalent to ±1.73 m/s^2^, equivalent to ±10° of tilt), this difference in gain may be attributable to the direction of the linear acceleration. However, it is more likely that a greater translation response is required during fore-aft translation to overcome the canal tilt cues. Another contributing factor for this difference could be related to cognitive aspects based on the expected motion paths – this argument is reinforced by the observation that the perception translation gain during fore-aft translation increased from one pre-flight session to the next [30].

Recently, we examined the modification of otolith-mediated responses during constant velocity OVAR after spaceflight [20]. This stimulus generates periodic linear acceleration along the interaural axis. As in the present study, perception reports were utilized to infer how the vestibular system resolves various motion stimuli. We observed that perception tilt gain increased at low frequencies (0.125 Hz) and perception translation gain increased at higher frequencies (0.5 Hz) post-flight relative to pre-flight. While we did not observe an increase in dynamic tilt gains at low frequencies in the present study, the increases in translation perception gains are consistent with the increased translation perception gain during OVAR [21].

One possible interpretation for these results is that perception of tilt in darkness becomes useless in weightlessness, as body tilt may not generate falls. On the other hand, the otoliths are still stimulated by body translation and by head rotation in pitch or roll. In fact, tilt-translation disturbances upon returning to Earth’s gravity reflect the adaptation to novel patterns of sensory cues experienced during motion on orbit. One of the most common post-flight illusions is of perceived translation, either of self or surround, during a tilting motion [9]. Approximately 90% of Space Shuttle astronauts reported self- and or surround motion illusions when performing ±20° sinusoidal head movements in pitch, roll, and yaw at 0.25 Hz within the first 3 hours post-flight [31]. A previous pre- and post-flight experiment had used a parallel swing to elicit roll-tilt and translation stimuli at 0.26 Hz, i.e. around the tilt-translation crossover frequency region. Six astronauts reported an increase in perceived lateral translation during passive roll rotation in darkness post-flight [18]. Decreased postural stability is also commonly observed after spaceflight [32].

One possible hypothesis for these changes is that of an otolith-tilt-translation reinterpretation based on the premise that interpretation of the otolith input as tilt is inappropriate during spaceflight. Therefore, during adaptation to weightlessness, the brain interprets otolith output to indicate translation [8], [9]. Merfeld [33] also suggested that certain neural processes of sensory integration adapt when astronauts experience weightlessness. These specific processes are those underlying the use of rotational cues to interpret ambiguous gravito-inertial cues via internal models [7], [34], [35]. An alternative hypothesis proposed by Guedry et al. [36] suggests that rather than a reinterpretation of otolith signals, adaptation to spaceflight might involve ‘shutting down’ the search for position signals from the otolith system in order to avoid vestibular conflict. The absence of a meaningful initial position signal from the otoliths on orbit may be eventually neglected, therefore increasing perception translation gain.

## Conclusion

The findings of this study confirm previous results documenting an overall increased motion sensitivity following short-duration spaceflight. During static tilt stimuli, an overestimation of roll tilt perception was observed on the day of landing. During dynamic linear acceleration stimuli along the X- or Y-axes, subjects reported larger sense of translation after the flight compared to before the flight. These changes are frequency dependent, with a lowering of the crossover frequency at which translation perception dominates. While the static tilt perception changes recover with the first day following short-duration spaceflights, changes in the dynamic responses persist longer, especially in the pitch plane.

Motion cues are known to be important in manual control of various aircraft (e.g., [37]). The alterations in motion perception demonstrated in this study are consistent with other changes in sensorimotor function that have been documented, including disruption to balance, locomotion, gaze control, dynamic visual acuity, and eye-hand coordination [25]. These alterations in sensorimotor function affect fundamental skills required for piloting and landing airplanes and space vehicles, driving automobiles and rovers, and operating remote manipulators and other complex systems [38]. This potential risk involving vehicular control will be even greater during long duration missions, e.g., during human asteroid and Mars missions. Sensorimotor disruption with aging and pathology are also likely to challenge the CNS resolution of sensory ambiguity and have operational consequences related to fall risk and vehicular control.
